# Utility, feasibility, and socio-demographic considerations in the diagnosis of bacterial RTI's by GC-IMS breath analysis

**DOI:** 10.1016/j.isci.2024.110610

**Published:** 2024-07-30

**Authors:** Trenton K. Stewart, Emma Brodrick, Matthew J. Reed, Andrea M. Collins, Emma Daulton, Emily Adams, Nicholas Feasey, Libbe Ratcliffe, Diane Exley, Stacy Todd, Nadja van Ginneken, Amandip Sahota, Graham Devereux, E.M. Williams, James A. Covington

**Affiliations:** 1 Warwick Medical School, University of Warwick, Coventry, UK; 2 School of Engineering, University of Warwick, Coventry, UK; 3 IMSPEX Diagnostics Ltd., Wales, UK; 4 Emergency Medicine Research Group Edinburgh (EMERGE), Royal Infirmary of Edinburgh, Edinburgh, UK; 5 Acute Care Edinburgh, Usher Institute, University of Edinburgh, Edinburgh, UK; 6 Clinical Sciences, Liverpool School of Tropical Medicine, Liverpool, UK; 7 Liverpool University Hospitals NHS Trust, Liverpool, UK; 8 NIHR CRN Northwest Coast, Liverpool, UK; 9 Brownlow Health, Liverpool, UK; 10 Department of Primary Care and Mental Health, University of Liverpool, Liverpool, UK; 11 Department of Infectious Diseases and HIV Medicine, University Hospitals of Leicester NHS Trust, Leicester, UK; 12 University of South Wales, Newport, UK

**Keywords:** Diagnostics, Chemistry, Analytical chemistry

## Abstract

Diagnosis of respiratory tract infections (RTIs), especially in primary care, is typically made on clinical features and in the absence of quick and reliable diagnostic tests. Even in secondary care, where diagnostic microbiology facilities are available, these tests take 24–48 h to provide an indication of the etiology. This multicentre study used a portable gas chromatography-ion mobility spectrometer (GC-IMS) for the diagnosis of bacterial RTIs. Breath samples taken from 570 participants with 149 clinically validated bacterial and 421 non-bacterial RTIs were analyzed to distinguish bacterial from non-bacterial RTIs. Through the integration of a sparse logistic regression model, we identified a moderate diagnostic accuracy of 0.73 (95% CI 0 · 69, 0 · 77) alongside a sensitivity of 0 · 85 (95% CI 0 · 79, 0 · 91) and a specificity of 0 · 55 (95% CI 0 · 50, 0 · 60). The GC-IMS diagnostic device provides a promising outlook in distinguishing bacterial from non-bacterial RTIs and was also favorably viewed by participants.

## Highlights

• A large multi-clinic breath analysis study for distinguishing bacterial RTI’s
• BreathSpec device distinguishes bacterial RTIs with moderate diagnostic accuracy
• Participant feedback and implementation of a user-centerd design
• Establishment of a potential non-invasive GC-IMS diagnostic device using breath

## Graphical abstract

**Figure:**
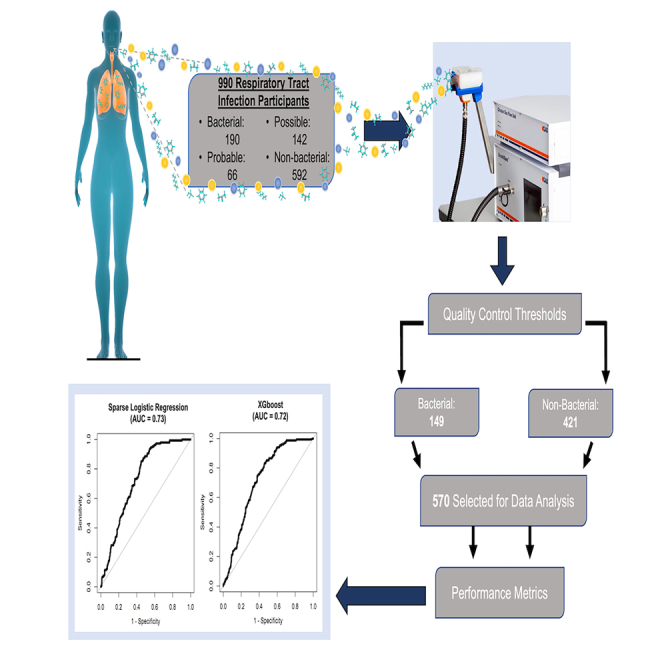

## Introduction

Worldwide, respiratory tract infections (RTIs) are a major cause of morbidity and mortality and the leading infection type in clinical medicine.^1^ These infections of the respiratory tract, include the sinuses, throat, airways, or lung parenchyma.^2^ While lower respiratory tract infections (LRTIs) affect the airways below the larynx including the lung tissue (parenchyma), upper respiratory tract infections (URTIs) occur in the structures in/above the larynx.^2^ These infections frequently have a viral etiology, especially in primary care. It is anticipated that rapid and accurate identification of the aetiologic agent of RTI will lead to a reduction in antibiotic prescribing.

Currently, there are few clinical diagnostic devices capable of distinguishing between infection types from exhaled breath,^3^ which in part, is due to the stringent sensitivity and specificity requirements outlined by the world’s regulatory authorities. The leading breath technologies capable of meeting these regulations are the electronic nose (e-nose), mass spectrometry (MS), and ion mobility spectrometry (IMS) devices. E-noses have the highest potential for miniaturization,^4^^,^^5^ but are limited by manufacturing consistency and their capability to preserve diagnostic integrity over time.^4^^,^^6^ MS-based devices, such as PTR-MS or SIFT-MS, have a wide range of detection capabilities and relatively unmatched selectivity and sensitivity in complex media, such as breath.^7^^,^^8^ Although promising, integration of MS-based devices into clinical practice has been slow due to limited automatic sample preparation, substantial technical expertise, and high operational complexity.^4^^,^^9^ All of which, could explain why only 5% of reported clinical tests utilize MS in clinical pathology, despite its high diagnostic capability.^10^ IMS is a well-established technique that has been shown to achieve equivalent sensitivity and selectivity as observed in MS-based devices. Yet, IMS-based devices can detect trace levels of VOCs without complicated pressure vacuums, as is required by most other devices with similar detection levels.^11^ IMS-based devices have begun to show their high potential to meet clinical requirements, while also addressing the current clinical market demands.^11^ To date, the use of GC-IMS as a diagnostic tool a variety of the diseases encompassing cancer,^12^^,^^13^^,^^14^ inflammatory bowel disease,^15^^,^^16^^,^^17^ COPD,^18^^,^^19^^,^^20^ and Alzheimer’s.^21^

The primary aim of this large, multicenter study was to investigate whether bacterial RTI could be distinguished from non-bacterial RTI in exhaled breath, utilizing a recently developed IMS-based diagnostic device. Only one previous study has been performed in this area, but this initial study was limited by its exploratory nature and small sample size of 71 subjects.^22^ This study will expand upon these initial findings reported in the pilot study. Moreover, previous research has demonstrated that demographic and clinical factors can be sources of bias and influence on diagnostic models.^23^ Our secondary aim was to understand the influence of demographic and clinical factors on the classification models. Lastly, as the GC-IMS breath devices were initially designed and strategically manufactured for this project, a participant feedback survey was implemented to assess the acceptability of the device and breath collection process.

## Results

990 participants were recruited, of which, 190 were considered to have met the gold standard criteria for bacterial RTI, while 592 were not deemed to meet this criterion. The introduction of quality control (QC) measures reduced the total participant numbers to 570, 149 (35%) of these were confirmed bacterial and 421 (65%) were confirmed as non-bacterial. A breakdown of participant distribution for each site after the QC measures is provided in Table 1.

**Table 1:** An outline of the number of participants alongside their certified diagnosis and after implementation of the quality control measures

Quality Controlled Dataset				
Secondary Care Sites		Bacterial	Non-Bacterial	Total
1	Leicester Royal Infirmary	16	39	55
2	Glenfield Hospital	17	22	39
3	NHS Lothian	4	7	11
4	CWM-TAF-Royal-Morgannwg Health Board	22	16	38
6	Royal Liverpool University Hospital	15	63	78
7	Aintree University Hospital	71	99	170
Primary Care Site				
8	Brownlow Health	4	174	178
Combined				
**-**	All	149	421	570

### Demographics

A breakdown of the clinical and demographic factors stratified by infection classifications is shown in Table 2.

**Table 2:** Clinical and demographic characteristics of the study participants grouped within the bacterial and non-bacterial classifications

	Bacterial	Non-Bacterial
**Participants**		

	149 (35%)	420 (65%)

**Clinical Factors**		

Infection Type		
Lower	69	133
Upper	6	181

**Demographic Factors**		

Sex		
Female	73	164
Male	76	254
Smoking Status		
Currently smoking	108 (72%)	193 (46%)
Weight (kg)		
Mean (SD)	72 (22)	75 (23)
Age		
1^st^ Quartile (25%)	53	21
Interquartile Range	67	42
3^rd^ Quartile (75%)	78	67
Ethnicity		
Asian	5	21
Black, Caribbean, or African	3	6
Caucasian	140	378
Mixed	0	0
Other	1	15

Abbreviations: SD = standard deviation; kg = kilogram.

### Diagnostic model outputs

#### Bacterial vs. non-bacterial

**Figure 1:**
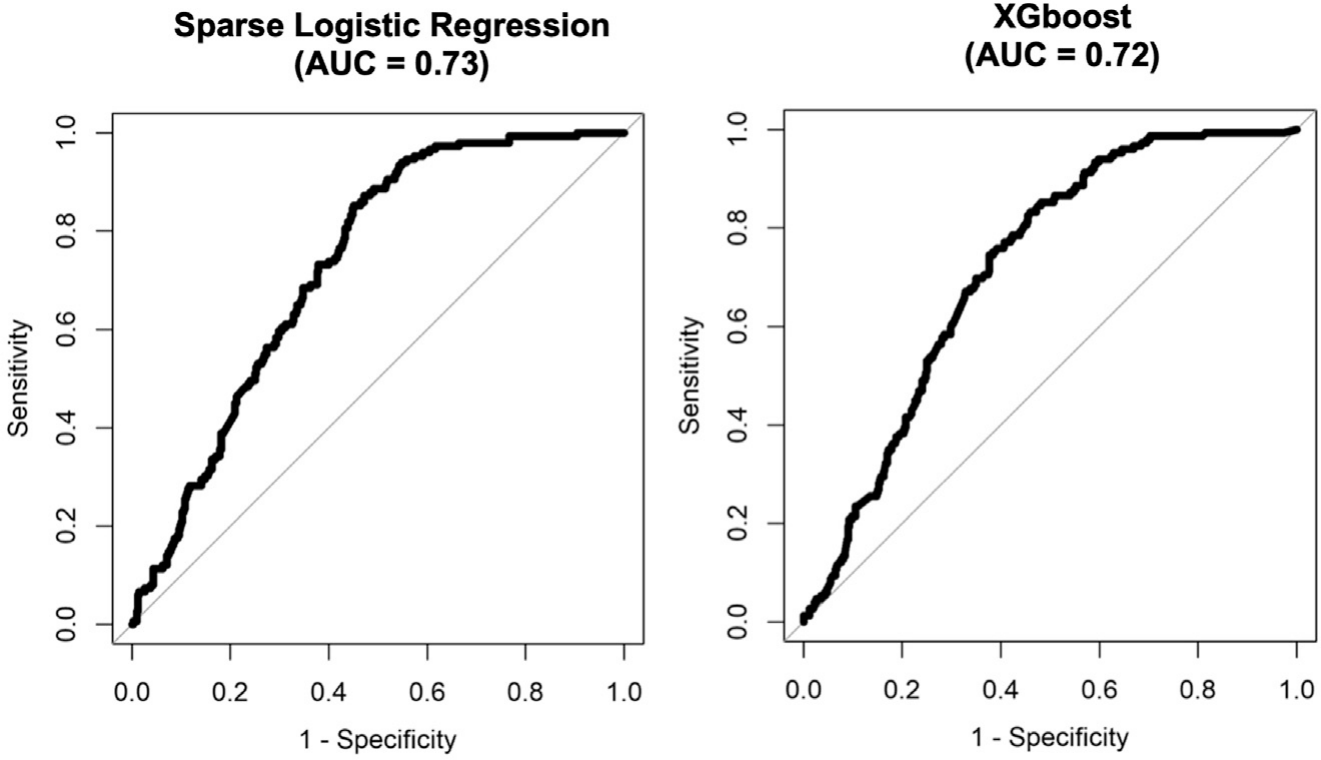
ROC curve plots produced from the sparse logistic regression and XGboost models with the resulting AUC output Abbreviations: ROC = receiver operating characteristic; AUC = area under the curve.

The primary focus of this study was to determine the diagnostic capability of the prototype BreathSpec device to distinguish between bacterial and non-bacterial RTIs, which achieved a diagnostic accuracy between 72 and 73%. In particular, the sparse logistic regression (SLR) model reported an area under the curve (AUC) of 0 · 73 (95% CI 0 · 69–0 · 77) with a sensitivity of 0 · 85 (95% CI 0 · 79–0 · 91) and a specificity of 0 · 55 (95% CI 0 · 50–0 · 60). The secondary model (XGboost) displayed an AUC result of 0 · 72 (95% CI 0 · 68–0 · 76) with a sensitivity of 0 · 83 (95% CI 0 · 76–0 · 89) and specificity of 0 · 54 (95% CI 0 · 49–0 · 59). Figure 1 displays the ROC plots for both the SLR and XGboost alongside their respective AUC outputs.

#### Confounding factors

In addition to the principal diagnostic output, several potential confounding factors were also screened. The results for all confounding factor analyses performed can be found in Tables 3 and 4, which are stratified by the analysis type.

**Table 3:** Result outputs obtained from the Sparse Logistic Regression and XGboost classifier models for each categorical comparison evaluated

Categorical Comparisons							
Factor	Classifier	AUC ±95%	Sensitivity	Specificity	PPV	NPV	P-value
Mean Age (>49Vs < 49)	SLR	0 · 81 (0 · 77–0 · 85)	0 · 88 (0 · 83–0 · 91)	0 · 65 (0 · 59–0 · 71)	0 · 74	0 · 82	2 · 97x10^−37^
	XG	0 · 80 (0 · 76–0 · 84)	0 · 96 (0 · 93–0 · 98)	0 · 59 (0 · 53–0 · 65)	0 · 72	0 · 93	7 · 28x10^−36^
At-Risk Age (>65Vs < 65)	SLR	0 · 77 (0 · 74–0 · 81)	0 · 91 (0 · 86–0 · 95)	0 · 56 (0 · 51–0 · 61)	0 · 50	0 · 93	1 · 53x10^−26^
	XG	0 · 73 (0 · 69–0 · 77)	0 · 94 (0 · 89–0 · 97)	0 · 51 (0 · 45–0 · 56)	0 · 48	0 · 94	7 · 22x10^−20^
Sex (Male Vs. Female)	GP	0 · 50 (0 · 45–0 · 55)	0 · 89 (0 · 85–0 · 92)	0 · 18 (0 · 13–0 · 23)	0 · 60	0 · 53	0 · 500
	XG	0 · 50 (0 · 45–0 · 55)	0 · 39 (0 · 34–0 · 44)	0 · 66 (0 · 59–0 · 72)	0 · 61	0 · 44	0 · 549
Sex (Female) & Age (>49Vs < 49)	SLR	0 · 76 (0 · 71–0 · 82)	0 · 90 (0 · 82–0 · 95)	0 · 58 (0 · 51–0 · 66)	0 · 56	0 · 91	4 · 78x10^−14^
	XG	0 · 77 (0 · 72–0 · 83)	0 · 94 (0 · 88–0 · 98)	0 · 54 (0 · 47–0 · 61)	0 · 54	0 · 94	3 · 89x10^−15^
Sex (Male) & Age (>49Vs < 49)	SLR	0 · 81 (0 · 74–0 · 87)	0 · 94 (0 · 90–0 · 97)	0 · 68 (0 · 57–0 · 78)	0 · 87	0 · 84	2 · 77x10^−16^
	XG	0 · 81 (0 · 74–0 · 88)	0 · 99 (0 · 96–1 · 00)	0 · 62 (0 · 51–0 · 72)	0 · 86	0 · 96	5 · 16x10^−17^
Smoking (Smoker Vs. Not)	SLR	0 · 70 (0 · 66–0 · 75)	0 · 89 (0 · 85–0 · 93)	0 · 56 (0 · 49–0 · 62)	0 · 69	0 · 82	2 · 20x10^−17^
	XG	0 · 71 (0 · 67–0 · 76)	0 · 90 (0 · 86–0 · 93)	0 · 53 (0 · 47–0 · 59)	0 · 68	0 · 83	5 · 41x10^−19^
Smokers & Sex (Male Vs. Female)	SLR	0 · 53 (0 · 46–0 · 59)	0 · 62 (0 · 54–0 · 69)	0 · 50 (0 · 41–0 · 59)	0 · 60	0 · 52	0 · 77
	XG	0 · 58 (0 · 52–0 · 65)	0 · 70 (0 · 62–0 · 77)	0 · 46 (0 · 37–0 · 54)	0 · 61	0 · 55	0 · 993
Non-Smokers & Sex (Male Vs. Female)	SLR	0 · 53 (0 · 46–0 · 61)	0 · 69 (0 · 61–0 · 76)	0 · 45 (0 · 35–0 · 54)	0 · 67	0 · 47	0 · 170
	XG	0 · 50 (0 · 43–0 · 58)	0 · 62 (0 · 55–0 · 70)	0 · 46 (0 · 36–0 · 58)	0 · 65	0 · 43	0 · 473
Age (>65) &Smoking (Smoker Vs. Not)	SLR	0 · 52 (0 · 41–0 · 63)	0 · 68 (0 · 59–0 · 75)	0 · 49 (0 · 32–0 · 66)	0 · 84	0 · 27	0 · 672
	XG	0 · 51 (0 · 41–0 · 62)	0 · 53 (0 · 44–0 · 61)	0 · 57 (0 · 39–0 · 73)	0 · 83	0 · 23	0 · 395
Age (<65) &Smoking (Smoker Vs. Not)	SLR	0 · 76 (0 · 72–0 · 81)	0 · 85 (0 · 78–0 · 9)	0 · 61 (0 · 54–0 · 67)	0 · 60	0 · 85	5 · 47x10^−19^
	XG	0 · 75 (0 · 70–0 · 80)	0 · 82 (0 · 75–0 · 88)	0 · 61 (0 · 54–0 · 67)	0 · 59	0 · 83	3 · 21x10^−17^

Abbreviations: SLR = Sparse Logistic Regression; XG = XGboost; AUC = Area Under the Curve; PPV = Positive Prediction Value; NPV = Negative Prediction Value.

**Table 4:** Result outputs obtained from the Sparse Logistic Regression and XGboost classifier models for each bacterial characterization factor evaluated

Bacterial Characterization **(Bacterial Vs. Non-Bacterial)**							
Factor	Classifier	AUC ±95%	Sensitivity	Specificity	PPV	NPV	P-value
Non-Smokers	SLR	0 · 80 (0 · 74–0 · 87)	0 · 93 (0 · 80–0 · 98)	0 · 71 (0 · 65–0 · 77)	0 · 37	0 · 98	3 · 72x10^−10^
	XG	0 · 73 (0 · 66–0 · 80)	0 · 95 (0 · 83–0 · 99)	0 · 52 (0 · 46–0 · 59)	0 · 27	0 · 98	1 · 75x10^−6^
Smokers	SLR	0 · 57 (0 · 51–0 · 64)	0 · 30 (0 · 21–0 · 39)	0 · 85 (0 · 79–0 · 90)	0 · 52	0 · 68	0 · 016
	XG	0 · 61 (0 · 54–0 · 68)	0 · 45 (0 · 36–0 · 55)	0 · 77 (0 · 71–0 · 83)	0 · 53	0 · 72	7 · 89x10^−4^
Under 65	SLR	0 · 77 (0 · 71–0 · 82)	0 · 92 (0 · 83–0 · 97)	0 · 60 (0 · 54–0 · 66)	0 · 36	0 · 97	2 · 16x10^−13^
	XG	0 · 76 (0 · 70–0 · 81)	0 · 91 (0 · 82–0 · 96)	0 · 54 (0 · 48–0 · 59)	0 · 32	0 · 96	2 · 83x10^−12^
Over 65	SLR	0 · 51 (0 · 43–0 · 60)	0 · 44 (0 · 32–0 · 55)	0 · 65 (0 · 56–0 · 74)	0 · 48	0 · 61	0 · 62
	XG	0 · 51 (0 · 43–0 · 60)	0 · 31 (0 · 21–0 · 42)	0 · 80 (0 · 72–0 · 87)	0 · 53	0 · 61	0 · 39
Sex (Female)	SLR	0 · 66 (0 · 60–0 · 72)	0 · 95 (0 · 87–0 · 99)	0 · 42 (0 · 36–0 · 48)	0 · 33	0 · 96	1 · 67x10^−5^
	XG	0 · 66 (0 · 60–0 · 72)	0 · 86 (0 · 76–0 · 93)	0 · 52 (0 · 46–0 · 59)	0 · 35	0 · 92	1 · 04x10^−5^
Sex (Male)	SLR	0 · 69 (0 · 62–0 · 76)	0 · 93 (0 · 85–0 · 98)	0 · 41 (0 · 33–0 · 49)	0 · 41	0 · 93	9 · 33x10^−7^
	XG	0 · 66 (0 · 58–0 · 73)	0 · 95 (0 · 87–0 · 98)	0 · 31 (0 · 24–0 · 39)	0 · 38	0 · 93	9 · 33x10^−5^
Sex (Female) & Non-Smokers	SLR	0 · 62 (0 · 47–0 · 76)	0 · 45 (0 · 23–0 · 68)	0 · 88 (0 · 81–0 · 92)	0 · 33	0 · 92	0 · 047
	XG	0 · 61 (0 · 48–0 · 73)	0 · 8 (0 · 56–0 · 94)	0 · 42 (0 · 34–0 · 50)	0 · 16	0 · 94	0 · 052
Sex (Female) & Smokers	SLR	0 · 57 (0 · 47–0 · 66)	0 · 43 (0 · 30–0 · 57)	0 · 73 (0 · 64–0 · 81)	0 · 45	0 · 71	0 · 085
	XG	0 · 59 (0 · 49–0 · 68)	0 · 29 (0 · 17–0 · 42)	0 · 89 (0 · 82–0 · 94)	0 · 57	0 · 71	0 · 031
Sex (Male) & Non-Smokers	SLR	0 · 64 (0 · 52–0 · 76)	0 · 62 (0 · 38–0 · 82)	0 · 68 (0 · 56–0 · 78)	0 · 33	0 · 87	0 · 023
	XG	0 · 74 (0 · 63–0 · 84)	0 · 81 (0 · 58–0 · 95)	0 · 69 (0 · 57–0 · 79)	0 · 40	0 · 93	4 · 8 x10^−4^
Sex (Male) & Smokers	SLR	0 · 66 (0 · 56–0 · 75)	0 · 54 (0 · 39–0 · 68)	0 · 75 (0 · 64–0 · 84)	0 · 57	0 · 72	1 · 03 x10^−3^
	XG	0 · 63 (0 · 53–0 · 74)	0 · 48 (0 · 34–0 · 62)	0 · 83 (0 · 74–0 · 91)	0 · 64	0 · 72	4 · 36 x10^−3^

Abbreviations: SLR = Sparse Logistic Regression; XG = XGboost; AUC = Area Under the Curve; PPV = Positive Prediction Value; NPV = Negative Prediction Value.

##### Age

Two age-related demographic factors were screened: mean and At-risk age. In the mean-age analysis screen, a high distinguishing accuracy and sensitivity was shown in both models. SLR reported an AUC of 0 · 81 (95% CI 0 · 77–0 · 85) and a sensitivity of 0 · 88 (95% CI 0 · 83–0 · 91). XGboost similarly reported an AUC of 0 · 80 (95% CI 0 · 76–0 · 84), but a much higher sensitivity of 0 · 96 (95% CI 0 · 93–0 · 98). The specificity for both models was moderate to low with SLR reporting 0 · 65 (95% CI 0 · 59–0 · 71) and XGboost at 0 · 59 (95% CI 0 · 53–0 · 65).

The second factor, at-risk age, reported a moderate AUC of 0 · 77 (95% CI 0 · 74–0 · 81) in the SLR model, while XGboost reported 0 · 73 (95% CI 0 · 69–0 · 77). Both models reported high sensitivities between 0 · 91–0·94 but had low specificities of just 0 · 51–0·56 (Table 3). When further merged with the smoking status classification, a difference could not be distinguished between smokers and non-smokers above the age of 65. Yet, a moderate diagnostic accuracy was obtained when differentiating between those below 65 years of age (Table 3). When stratified by bacterial infections, a moderate diagnostic accuracy was reported at 0 · 77 (95% CI 0 · 71–0 · 82) and 0 · 76 (95% CI 0 · 70–0 · 81) for XGboost in participants under 65 years of age. However, the diagnostic accuracy was substantially reduced when screening those over 65 years of age, where both models reported an AUC of just 0 · 51 (95% CI 0 · 43–0 · 60) (Table 4). A prominent difference in sensitivity was also observed when comparing the at-risk age groups, in which, the over 65 group reported a sensitivity value of 0 · 92 (95% CI 0 · 83–0 · 97) while the under 65 group reported a value of only 0 · 44 (95% CI 0 · 83–0 · 97).

##### Smoking status

53% of the patients stated they were smokers. Of those diagnosed with a bacterial RTI, 19% were smokers and 7% were non-smokers. A diagnostic accuracy of 0 · 70 (95% Cl 0 · 66–0 · 76) was reported when distinguishing smokers from non-smokers (Table 3). When stratified into the bacterial classification, non-smokers reported a moderate diagnostic accuracy of 0 · 80 (95% CI 0 · 74–0 · 87) for the SLR model and 0 · 73 (95% CI 0 · 66–0 · 80) for XGboost. However, this was not observed for smokers which reported an AUC of 0 · 57 (95% CI 0 · 51–0 · 64) and 0 · 61 (95% CI 0 · 54–0 · 68) for XGboost in the SLR model (Table 4).

### Feature overlay plots

**Figure 2:**
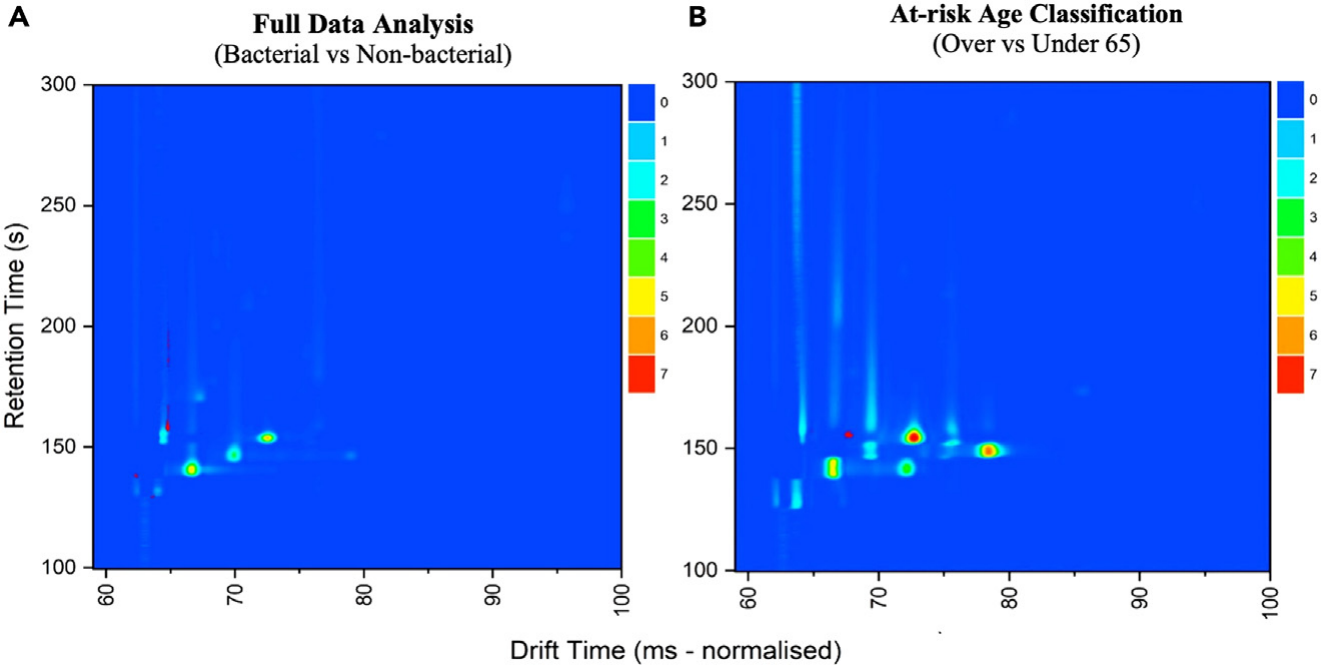
An overlay of the discriminatory features established in two separate analyses (A and B) onto designated topographic plots The y axis is the retention time obtained from the gas chromatography column and the x axis is the drift time reported by the ion mobility spectrometer.

Once the confounding factors were screened, the diagnostic features were overlayed onto topographic plots to identify areas of interest and compare feature locations. As can be observed in Figure 2A, two prominent feature locations were identified (outlined in red) when comparing bacterial and non-bacterial participants. The feature locations were predominantly contained between a drift time (DT) of 62–64 milliseconds (ms) and a retention time (RT) of 130–240 seconds (s). The age discriminatory features were isolated to an area with approximately 68ms DT and 155s RT (Figure 2B). Although not shown in the reference sample designated for this overlay plot, there is an apparent discriminatory peak at this location indicative by the circular concentration of features.

### Subject questionnaire

**Figure 3:**
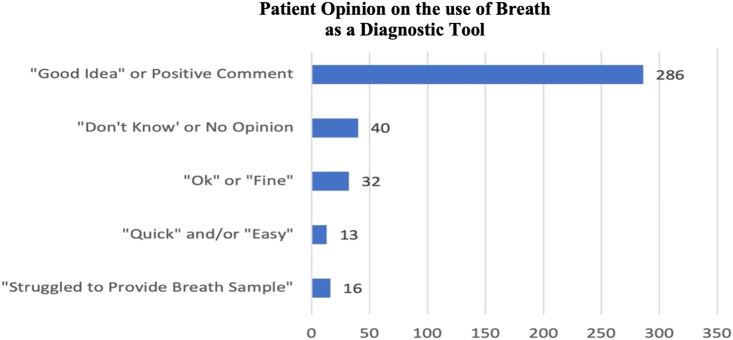
A summary and count of the subjects’ opinions on the breath collection process and BreathSpec device obtained from the participant questionnaire

A response rate (RR) of 49% was obtained for the participant questionnaire. 16 participants (4%) commented that they found it difficult to provide a breath sample. An expected response due to the need to obtain, primarily, the alveolar portion of the exhaled breath. Thus, requiring a deep exhalation, which can cause some discomfort and coughing in participants. Despite this exertion, participant opinions on the breath collection process and device were positive with over 73% of the comments indicating a positive comment or stating it was a “good idea”. Moreover, 32 participants (8 · 5%) found the implementation of breath was just “ok” or “fine”, while another 10% had no opinion on it. In summary, the use of breath as a diagnostic medium seems to be well received with only a few negative comments associated to the necessary long exhale (Figure 3).

**Figure 4:**
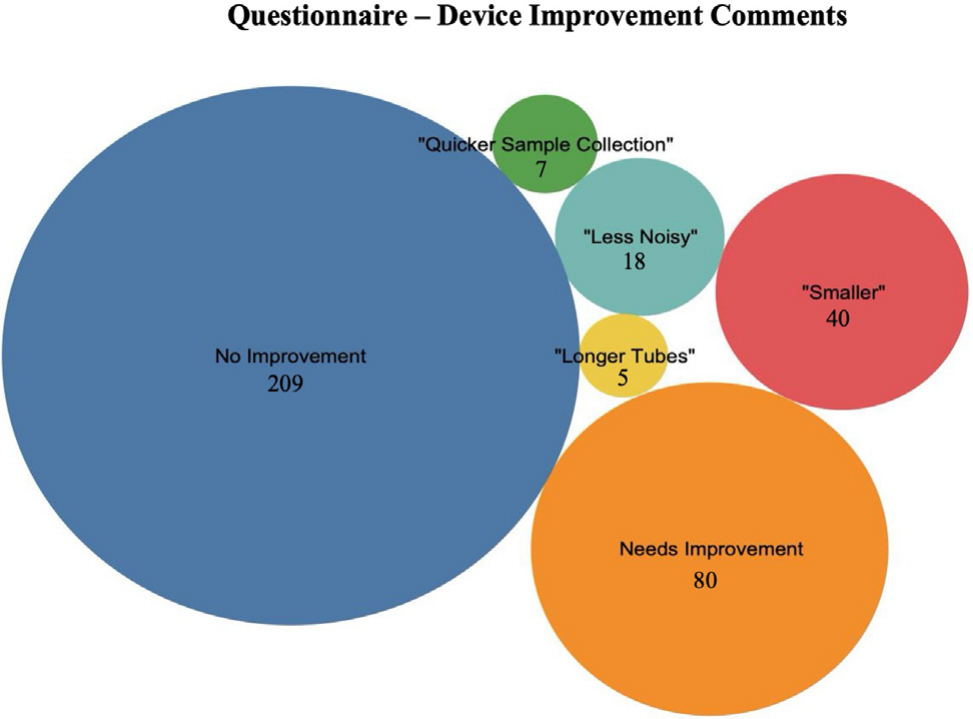
A clustered bubble chart depicting the comments provided in the participant questionnaire for the improvement of the BreathSpec device

A total of 289 participants provided feedback on the BreathSpec device (29% RR). Of these 289 participants, 209 (72%) stated that the device did not need improvement. An additional 80 stated the BreathSpec needed improvement, of which, 70 provided additional comments on the exact areas that could be developed; half of the comments suggested the device should be smaller, 22 · 5% requested it to be less noisy, roughly 9% wanted a quicker sample collection, and 6% wanted longer sample collection tubes (Figure 4).

## Discussion

In this study, GC-IMS based diagnostic devices were integrated into several clinical sites across the UK to analyze VOCs in the exhaled breath of participants presenting with symptoms of RTIs. Data analysis incorporated 570 RTI participants with 149 being clinically verified with a bacterial diagnosis and 421 as non-bacterial into two machine learning models. Both models reported moderately high and near identical diagnostic accuracies in distinguishing bacterial from non-bacterial RTIs (SLR model: 0 · 73; XGboost: 0 · 72). This study marks only the second study to have implemented GC-IMS technology to distinguish bacterial RTIs using breath. The initial pilot study conducted by Lewis et al.^22^, reported an AUC of 0 · 73, a sensitivity of 0 · 62, and a specificity of 0 · 80 when discriminating between bacterial and non-bacterial RTIs.^22^ In comparison, our study reported a higher sensitivity than were reported in the pilot (SLR: 0 · 85; XG: 0 · 83), but also had a reduction in the specificity (SLR: 0 · 55; XG: 0 · 54). The pilot study only incorporated 71 participants from a single clinical site, whereas our study was able to provide a sizable cohort from several clinical sites to substantiate the tentative results identified in the earlier study. This reduction could be attributed to the disproportionate number of participants classified as non-bacterial compared to bacterial, which has been known to influence specificity outputs in diagnostic models.^24^ Despite this, our study was able to report better sensitivities and similar diagnostic accuracies, despite the reduction in specificities, compared to the pilot study.

Furthermore, we speculated that a variety of clinical and socio-demographic factors were possible influencers affecting the diagnostic capability of the classifier models. To explore this further, age, and smoking status were screened. Age-related factors were indicated as probable influencers on the diagnostic accuracy (Table 3). When the at-risk group was further stratified by the bacterial classification, a moderate diagnostic accuracy was reported for participants below 65 years of age but was not shown for those above this age limit. This implies that analyzing samples from patients over 65 years old had a negative impact on the discriminatory capability of the models. This suggests that there are additional factors impacting the discernment capabilities for those above this age group. Several age-related VOCs have already been linked to patients above 65 years. For example, Cakmak et al. found an association between reduced lung function and three VOCs (hexanal, a-pinene, and 2-methyl-1,3-butadiene) in the elderly above 65 years of age.^25^ Altomare et al. also utilized a combination of 14 exhaled VOCs to discriminate patients with colorectal cancer from those without, specifically in patients above 65 years of age.^26^ The feature plots also indicated a third, unique, feature solely identified in only the >65 bacterial group classification. The area was concentrated within a peak located at DT 68ms and an RT of 170s (Figure S3B), suggesting that the intensity values associated to this peak could be an age-related discriminatory factor. Once participants over 65 years old were removed, a slight increase in AUC and sensitivity values were observed when compared to the full data bacterial classification. Altogether, these demonstrate the importance of addressing age factors in future breath studies.

Smoking status was screened as a potential influencing factor. The diagnostic models showed that smokers could be moderately distinguished from non-smokers in the dataset. When incorporated into the bacterial classification analysis, non-smokers reported an AUC of 0 · 80 when differentiating between bacterial and non-bacterial participants in the SLR model. The feature plots indicated the peak located at DT 64ms and an RT of 160s as the key distinguishing factor (Figure S4). Yet, an AUC of just 0 · 61 (SLR) was observed in the same analysis for smokers. In comparison to the results obtained from the full dataset, the removal of smokers increased the diagnostic accuracy and sensitivity. The reduction in diagnostic capability from smokers could be associated to the toxic and carcinogenic compounds inhaled when smoking, such as benzene, styrene, acetonitrile, and 2,5-dimethylfuran.^27^ This in turn, diminishes the potential diagnostic VOCs through saturation of these compounds in breath. Moreover, an increase in specificity was also reported solely in the SLR model from 0 · 55 (95% CI 0 · 50–0 · 60) in the full dataset to 0 · 71 (95% CI 0 · 65–0 · 77) in the non-smoker group. It has been established that cigarette smoke affects the host-metabolism through the increase of oxidative stress leading to the formation of free radicals and lung inflammation.^27^ In combination, these elements are likely to mask important VOCs in the breath and reduce diagnostic accuracy.

Finally, as the BreathSpec devices were strategically designed and manufactured for this project, we thought it was important to consider a patient’s perspective on providing a breath sample and on the diagnostic device itself. To achieve this, a user-centerd design was implemented, which has shown to lead to better quality products with lower error rates, while establishing efficient and effective devices that address the diverse needs of participants,^28^^,^^29^. This study reported a 49% RR for feedback associated to delivering a breath sample and a 29% RR on the device itself. Using this approach, key concerns could be addressed and improved in the next generation of BreathSpec devices. Over 22% of comments were associated to the noise of the device, primarily associated to the CGFU unit. Upgrades to the pumps were able to reduce this noise level. Although over half of the comments were associated to the size of the device, the initial size of the device was deliberately designed to be portable and is smaller than similar spectrometry-based devices. 6% of the comments were associated to the length of the sample collection tubes. It was determined that increasing the length of the tubes would also require a time-delay for the breath samples to enter the device. Thereby, adding to the analysis time and the potential to reduced sample input. This issue was addressed through the design of a new, remote, breath collection procedure. This procedure involves the integration of a short cylindrical tube with a syringe attached to the middle. As the participant exhales through the reservoir tube, the syringe is pulled down to collect the breath sample and then directly injected into the BreathSpec. Thus, removing the need for attached tubing or heavy handheld breath collection masks. This breath collection procedure was successfully implemented in a COVID-19 study by Nazareth, J. et al. (2022) and can be visualized in Figure S3 from the article.^30^ Notably, through the utilization of the next generation BreathSpec devices and breath collection procedure, they were able to discriminate COVID-19 patients from healthy controls with an AUC, sensitivity, and specificity of 0.85, 0.80, and 0.88.

### Limitations of the study

There are several limitations that must be considered in this study. To start, we were unable to investigate medical treatments, alcohol-use, or comorbidities as bias factors due to limited patient data. Although the breath collection procedure utilized in this study aimed to collect the last portion of the breath, CO_2_ concentrations in the exhaled breath were not measured to determine the exact end-tidal portion. Thus, the sample is still considered to be of mixed-breath origin. It is worth noting that participant recruitment was completed in 2018, before the COVID-19 outbreak, and the routine PCR testing for viral RTIs established during the pandemic had not been implemented at the time. Additionally, participants were only able to be recruited from a single primary care site and where there were only five definitive confirmed bacterial cases. This prevented the capability to analyze differences between primary and secondary care participants. The dataset also combined URTI and LRTI’s due to a low number of URTI classified into the bacterial infection group. This was due to the limited certainty that current diagnostic techniques were able to determine a bacterial URTI. It is possible these combinations influenced the diagnostic accuracy due to the differences in participant demographics and colonizing bacteria in the URT and LRT. Further studies will be required to distinguish the differences between these sites and URT/LRTI categorizations. Finally, this study utilized a data mining approach for data analyses that removed the need for *a priori* knowledge of the VOCs or their peak locations. This allowed the discriminatory features to be visualized by overlaying them onto the GC-IMS topographic plots to display areas of interest. However, this process limits the capability to establish a biological link and can only indicate areas-of-interest for potential diagnostic VOCs. Despite this, this methodology has also been shown to provide an efficient way to eliminate background information while preserving diagnostic accuracy.^31^^,^^32^

### Conclusion

This study was able to demonstrate the feasibility of implementing the BreathSpec, a portable GC-IMS breath device, in both primary and secondary care. It is only the second study to ever be conducted utilizing GC-IMS technology to discriminate this infection type and, to the best of our knowledge, the largest to date. As such, one of the core strengths of this study is the large cohort size and true population heterogeneity that was obtained through an unselected recruitment process at the clinical level. Although it is also believed that this heterogeneity was a contributing factor to the low specificity reported in our diagnostic models. A likely effect compounding socio-demographic factors, in addition to the population heterogeneity, influencing the detection of exhaled diagnostic VOCs. In which, this study was able to establish that both age and smoking status are key socio-demographic factors affecting diagnostic accuracy. Despite the low specificity, these first generation BreathSpec devices were able to still produce a moderate diagnostic accuracy and high sensitivity. Nonetheless, utilizing current diagnostic techniques, only 48% of participants with a bacterial RTI were classified as definitive and able to be utilized in this study; a prominent factor that influenced our sample size. This outcome, in of itself, can show that there is a critical need for more accurate and reliable diagnostic tests to distinguish bacterial RTIs in both primary and secondary care facilities. Moreover, currently implemented point-of-care tests, such as rapid antigen tests or lung ultrasounds, could be combined with the GC-IMS to provide quick, reliable, and improved diagnostic outputs than is observed individually. Either through this integration of existing techniques to the GC-IMS or through advancements in the BreathSpec, further studies will need to look at the diagnostic capability of the GC-IMS to predict RTIs over time and their potential to fill this gap and assist in the reduction of antibiotic overprescribing.

## STAR★Methods

### Key resources table

**Table:** 

REAGENT or RESOURCE	SOURCE	IDENTIFIER
**Software and algorithms**		

R (Version: 2022.07.1-Build 554)	R Foundation for Statistical Computing	http://www.R-project.org
Labview (Version 2023-Q1)	National Instruments	https://www.ni.com/en.html
OriginPro	Origin Lab	https://www.originlab.com/

**Other**		

BreathSpec	Imspex Diagnostics – G.A.S.	https://www.imspex.com/

### Resource availability

#### Lead contact

Further information and requests for resources should be directed to and will be fulfilled by the lead contact, Trenton Stewart (trenton.stewart@warwick.ac.uk).

#### Materials availability

This study did not generate new unique reagents.

#### Data and code availability

• All data reported in this paper will be shared by the lead contact upon request.
• This paper does not report original code.
• Any additional information required to reanalyse the data reported in this paper is available from the lead contact upon request.

### Experimental model and study participant details

This study recruited adult patients presenting in primary and secondary care with suspected upper and/or lower respiratory tract infections (URTI/LRTI). Following suspected infection, potential participants were invited to take part and study procedures were quickly followed in the respective clinical site. The study involved six secondary care (hospital) sites and one primary care (general practice) site within the UK. Recruitment occurred over a 7-month period from 2018 to 2019. The criteria for inclusion encompassed those ≥18 years of age and a clinical suspicion for upper and/or lower respiratory tract infection. Patients were excluded if they had received antibiotics within the previous 6 h, had a diagnosis of lung cancer, had received cancer chemotherapy within the previous 30 days, and/or a history/pre-existing diagnosis of iatrogenic neutropoenia. Potential participants were identified by clinical triage within the acute hospital setting (Emergency Department or acute medical wards), or from patients presenting for general practice review. Recruitment and study protocols and procedures were completed at initial presentation. UK ethical approval was granted by NHSREC (approval no.: 18/LO/1029) and all participants provided written informed consent.

In addition to breathe analysis and demographics the following routine clinical data were extracted from the clinical records, if available. In secondary care: routine observations, clinical examination findings, chest radiology, sputum culture, nose & throat swabs for viral and bacterial (including atypical bacterial) identification using standard culture methods and/or polymerase chain reaction (PCR), urinary pneumococcal/legionella antigens, routine hematology, biochemistry and CRP. In primary care: chest radiology, sputum culture; nose and throat swabs for viral and bacterial (including atypical bacterial) identification using standard culture methods and/or PCR testing. A breakdown of the number of recruited participants for each site alongside their classification is provided below (Table S1).

Based on clinical data, experienced clinicians utilised the following framework to classify participants into four categories.^33^^,^^34^

(1) Definite bacterial pneumonia
  a. New lobar or multi-lobar consolidation on chest radiograph or CT.
(2) Probable bacterial pneumonia
  a. Radiological imaging suggestive (but not diagnostic), AND positive microbiology (sputum positive OR swab PCR positive for respiratory bacterial pathogen).
  b. Imaging suggestive (but not diagnostic), AND CRP ≥100 +/− clinical examination in keeping with pneumonia BUT microbiology negative/not taken.
  c. Imaging unremarkable, BUT CRP ≥100 AND clinical examination suggestive AND positive microbiology.
(3) Possible bacterial pneumonia
  a. Imaging suggestive (but not diagnostic), AND clinical examination in keeping with pneumonia BUT microbiology negative/not taken.
  b. Imaging unremarkable BUT Positive microbiology AND CRP between 20 and 100.
  c. Imaging unremarkable BUT Positive microbiology AND clinical examination in keeping with pneumonia AND CRP between 20 and 100
  d. Imaging unremarkable AND microbiology negative/not taken BUT clinical examination suggestive of pneumonia AND CRP ≥100.
(4) Not bacterial pneumonia
  a. Imaging suggestive, (but not diagnostic) OR normal AND Negative microbiology AND CRP <20.
  b. Imaging unremarkable AND microbiology negative/not taken.

#### Sample size calculation

This study aimed to discriminate between bacterial and non-bacterial RTIs through the implementation of AUROC curves as a measure of diagnostic accuracy. To ensure statistical relevance, an intial sample size calculation was performed. As the true prevalence and diagnostic accuracy were unknown at the start of this study, a single-test design against a null value was utilised for the calculation; as outlined in Akoglu, H (2022).^35^ The prevalence for bacterial RTIs was set to 50% with a power of 90% and a confidence level of 95%. The pilot study conducted by Lewis et al. *(2017)*^22^ provided the sensitivity and specificity estimate values. As such, a potential sensitivity (seP1) of 60% and a potential specificity (spP1) of 80% were chosen. The null values (P0) were set at 50% for sensitivity and 70% for specificity. This calculation identified a total sample size required to estimate a true prevalence of RTI bacterial at 556 participants (with Yates’ Continuity Correction). However, as the pilot study only contained 12 confirmed viral and 7 confirmed bacterial RTI’s, we decided it was necessary to perform an additional sample size calculation post-factum with larger participant pool. To ensure similarity, the diagnostic performance reported by Nazareth et al. (2022) utilising a GC-IMS instrument to distinguish COVID-19 patients from other RTIs in breath was selected.^36^ This study reported a sensitivity of 80% and a specificity of 90%.^36^ These were selected as the null values and the spP1 and seP1 were set as similar to the values reported by Lewis & colleagues. This calculation identified a total sample size necessary to estimate the true prevalence of bacterial RTIs at 281 participants (with Yates’ Continuity Correction).

### Method details

#### Breath sample collection

Prior to breathe collection, the participant was asked not to eat or drink for 30 min and to rinse out their mouth with water. For collection, the participant was asked to take a deep breath, but not to hold it, and then breathe out normally through a disposable mouthpiece, into a reservoir tube, for as long as they could. The breath sample was then drawn directly into the machine from the reservoir tube (Figure S1) and into the instrument containing a 1 mL sample loop for immediate analysis. For note, the sample would be taken from the last portion of the breath to ensure the breath used for analysis was primarily a mixed breath of end-tidal or alveolar portion, which is considered to have the highest concentration of the VOC chemical information from the lungs and bloodstream.^34^ The combination of the sample loop and delayed breath collection excludes the possibility of a dead volume being collected. Directly after the participant’s breath was analyzed, a room air sample was introduced into the instrument to check the background for air contamination before the data analysis stage. Breath collection and GC-IMS analysis took 10 min for each participant.

#### Gas chromatography-ion mobility spectrometry

This study manufactured the first generation BreathSpec (G.A.S., Dortmund, Germany) devices, a GC-IMS-based diagnostic platform designed to separate and detect trace VOCs in complex exhaled breath samples.^37^ The platform utilises a 2-fold separation strategy through the implementation of gas chromatography alongside Ion Mobility Spectrometry. The detection limits of GC-IMS devices have been established to have similar detection limits as to those observed in GC-MS. However, due to the necessity to form proton clusters, the detection limits for VOCs will be dependent upon their proton affinity. For example, typical detection limits for polar and medium polar VOCs, such as alcohols or ketones, range in the lower ppb to upper ppt. Nonpolar species, such as alkanes, range from the mid to upper ppb levels.^38^^,^^39^ The samples were tested using the following instrument settings; drift gas flow rate of 150 mL/min, carrier gas flow of 15 mL/min. Thereafter, the flow rate was ramped to 50 mL/min over the 10-min analysis time. The temperatures of the sample loop, transfer line, GC column, and IMS segments were set to: Sample loop: 55°C, Transfer line: 50°C, GC column: 40°C, and IMS: 70°C.

### Quantification and statistical analysis

#### Classifications

The study data analysis only included the Definite (Bacterial) and Not Detected (Non-Bacterial) RTI participants to optimise model accuracy by removing possible false positives. Smoking status was determined by the participant declaring they were a current smoker and non-smokers were any participants who had either never smoked or did not identify as a current smoker. Two age-related demographic factors were screened: Mean and At-risk age. Mean age was calculated by averaging the ages from all participants in dataset (Mean: 49.32; SD: 23.91). At-risk age was chosen due to the significantly higher mortality and morbidity rates in respiratory infections identified for those above the age of 65 years.^31^^,^^35^^,^^36^ Furthermore, this study combined both upper and lower RTIs within the respective classifications (bacterial vs. non-bacterial). This was due to a limited number of bacterial subjects diagnosed with an upper RTI.

Demographic factors were first separated into groups and then screened through the supervised feature selection process. The datasets were classified into two comparison groups for analysis: categorical and bacterial characterizations. The prior was implemented first to identify whether the models could separate clinical or demographic factors from the data (i.e., smoker vs. non-smoker). Thereafter, the bacterial characterization isolated the bacterial and nonbacterial subjects within each clinical or demographic classification (i.e., smoker – bacterial vs. non-bacterial).

#### Quality control measures

The two-factor categorized dataset was then screened against several Quality Control (QC) parameters. To start, the entire dataset was manually validated to ensure that none of the data were corrupted and that the spectra obtained during breath collection was viable. This check, alongside the exclusion of both the probable and possible categorizations, reduced the total number of participants to 782. Once the data validation had been performed, a series of pre-processing steps were included to reduce the dimensionality and align the BreathSpec spectral outputs. This is necessary due to the high dimensionality of the spectra, which can contain over 11 million data points. A description of the pre-processing steps can be found in Emma Daulton et al. (2021).^40^ Next, there were small variances in retention and drift time between instruments, due to manufacturing variances. To account for this, the spectral outputs were aligned utilising a custom alignment software. This software utilises a reference spectrum alongside the relative position of the RIP peak to align all spectra. Once completed, the software provides a mean square alignment error value (MSV) from the reference spectra. An MSV threshold was applied to the data to account for significant alignment variations that could influence the functionality of the diagnostic models. A moderate MSV threshold of 0 · 035 was applied to the data to limit large deviations while providing the necessary leeway to not remove viable data.

Finally, one of the key concerns in breath collection is the discernment between a genuine breath sample and the surrounding room. To verify this, acetone was used as a reference VOC, as it is well-established to be both consistent and stable in the human breath and has a concentration well above the detection limits of the BreathSpec. An acetone reference standard was analyzed to identify the range of the acetone peak on the BreathSpec spectral output. A box range for this peak was defined using VoCAL (G.A.S., Dortmund, Germany; version 0.1.3) software and the value of the highest intensity datapoint, within this range, was identified. The peak intensity value for acetone, which is linked to the rescaled RIP height, was obtained for every participant sample. The dataset was then split in half based on the intensity values and all the lower half spectral outputs were compared to establish a threshold that could be applied to the dataset to remove room air samples without eliminating viable samples. In consequence, any participant sample below this established threshold of 500, was removed from the analysis and considered a room air sample. An overview of the entire recruitment, QC measures, and data analysis is shown in Figure S2.

#### Statistical analysis

In completion of the pre-processing and QC steps, the final dataset was then implemented into a supervised feature selection process in ‘R’ (R Foundation for Statistical Computing, Vienna, Austria; version 2022 · 07·1 – Build 554). This method performs binary class prediction using a *k*-fold cross validation methodology that separates the dataset into ten equally sized subsets. Thereafter, a single subset was used for validation and the other nine were used to train the data (90:10 split), this is repeated until all the data have been used as a test sample. Features were identified through the implementation of a Wilcoxon rank-sum test onto the defined diagnostic groups. This process only occurred with the training set to remove data leakage from the test and train sets. It is important to note that the identified features are obtained solely on a statistical basis and do not have any biological significance. The top 100 features with the lowest *p*-value are identified and implemented into two separate classifier models. The models and their respective ‘R’ packages were: Sparse Logistic Regression (SLR) – glmnet (version 4.1–6) and XGboost (XG) – XGboost (version 1.6.0.1). This approach provides statistically significant and compact datapoint features that can be integrated into machine learning algorithms to train classifier models.^31^ From the resultant probabilities obtained from the models, statistical performance values were calculated. These encompassed the sensitivity, specificity, positive predictive values (PPV), negative predictive values (NPV), and the *p*-value outputs for every diagnostic analysis. The results from each of the diagnostic models was exhibited with a Receiver Operating Characteristic (ROC) curve and an Area Under the Curve (AUC) value was produced to report diagnostic accuracy.

#### Feature plotting

A visualisation of the identified features onto participant spectral outputs were obtained through the implementation of a custom feature integration platform using the software LabVIEW (National Instruments, Austin, Texas, USA; version 2023-Q1) to integrate the extracted features onto a designated spectral output within the equivalent analysis. The resulting output was then transformed into a matrix with the X axis corresponding to the drift time and the Y axis referencing the GC retention time. The X axis of the matrix was normalised, utilising the normalisation feature in the OriginPro (OriginLab, Northampton, Massachusetts, USA; version 2023–10.0), and a heatmap map was produced from the matrix.
