# Supplementary material for: Utility, feasibility, and socio-demographic considerations in the diagnosis of bacterial RTI's by GC-IMS breath analysis
Source: iScience. 2024 Jul 30;27(9):110610. doi: 10.1016/j.isci.2024.110610 (PMC11388771; doi:10.1016/j.isci.2024.110610)
Supplement: Document S1. Figures S1–S4 and Table S1 [file mmc1.pdf]

iScience, Volume *27*

Supplemental information

Utility, feasibility, and socio-demographic considerations in the diagnosis of bacterial RTI's by GC-IMS breath analysis

Trenton K. Stewart, Emma Brodrick, Matthew J. Reed, Andrea M. Collins, Emma Daulton, Emily Adams, Nicholas Feasey, Libbe Ratcliffe, Diane Exley, Stacy Todd, Nadja van Ginneken, Amandip Sahota, Graham Devereux, E.M. Williams, and James A. Covington

Supplementary Table 1: An outline of the number of participants alongside their certified diagnosis and after implementation of the quality control measures– Related to STAR Methods

Dataset Site Breakdown						
**Secondary Care Sites**		**Definite**	**Not Detected**	**Possible**	**Probable**	**Total**
1	Leicester Royal Infirmary	20	55	13	7	95
2	Glenfield Hospital	21	30	1	13	65
3	NHS Lothian	23	55	12	1	91
4	CWM-TAF-Royal-Morgannwg Health Board	23	21	10	17	71
6	Royal Liverpool University Hospital	23	97	46	9	175
7	Aintree University Hospital	75	122	21	15	233
Primary Care Site						
**8**	Brownlow Health	5	212	39	4	260
Combined						
**-**	All	190	592	142	66	990

Footnote: A manufacturing error occurred at the Princess Park Health Centre causing a loss of data and reliability of data. Therefore, this site was not able to be utilised and removed from the study.

Supplementary Figure 1: Breath Sample Collection – Related to STAR Methods

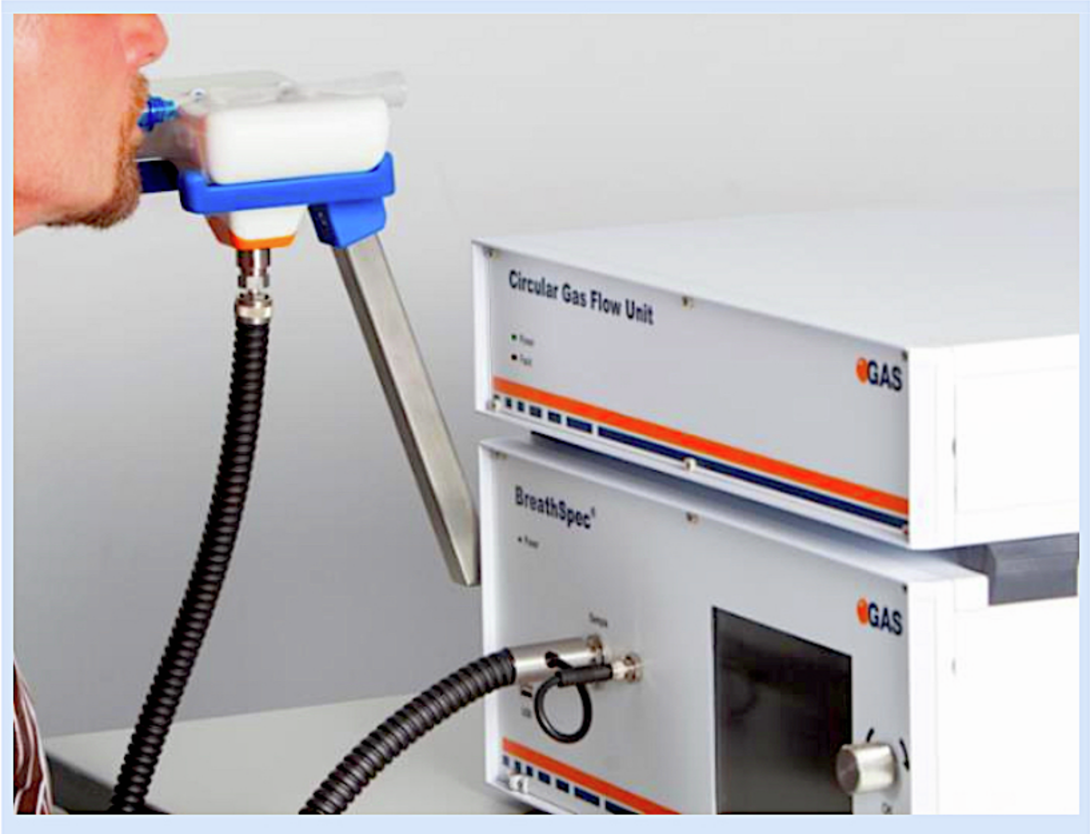

Supplementary Figure 1: An image depicting the set-up for the administration of a breath sample into the BreathSpec diagnostic device.

Supplementary Figure 2: Data Analysis Outline – Related to STAR Methods

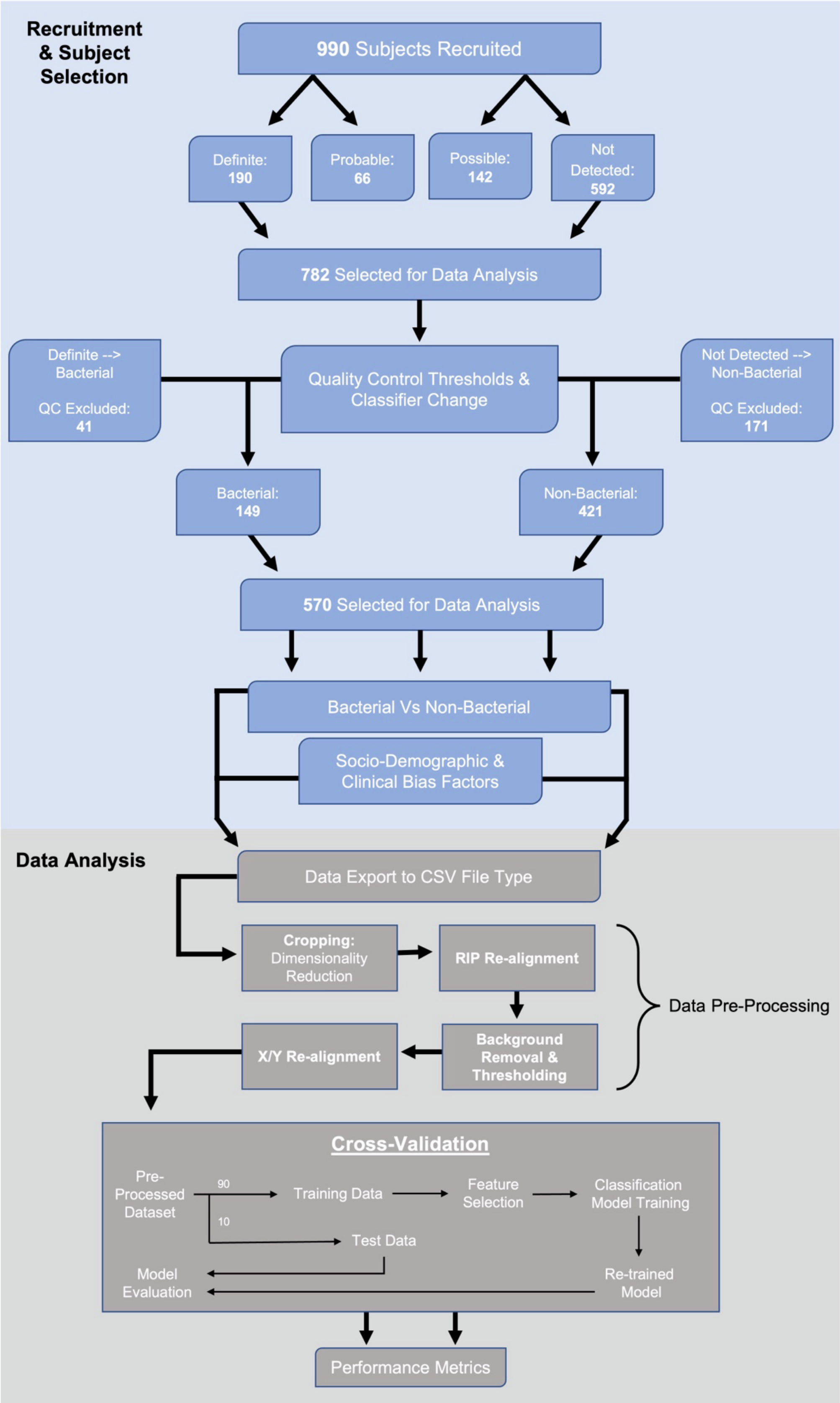

Supplementary Figure 2: A generalised overview of the recruitment, quality control measures, and data analysis on the RTI dataset. Abbreviations: RIP = Reactant Ion Peak.

Supplementary Figure 3: Identified age-related features – Related to Figure 4

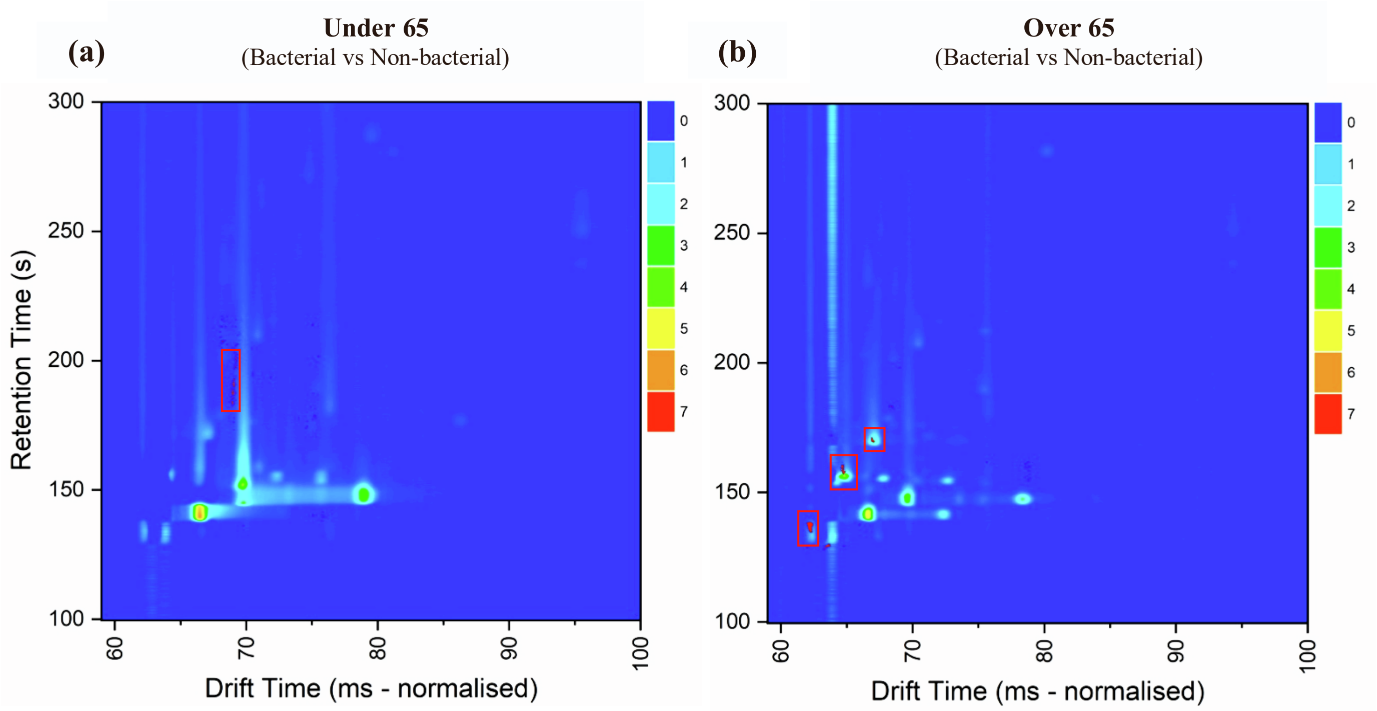

Supplementary Figure 3: An overlay of the discriminatory features established in two separate analyses (a-b) onto designated topographic plots. The y-axis is the retention time obtained from the gas chromatography column and the x-axis is the drift time reported by the ion mobility spectrometer.

Supplementary Figure 4: Identified features for smoking – Related to figure 4

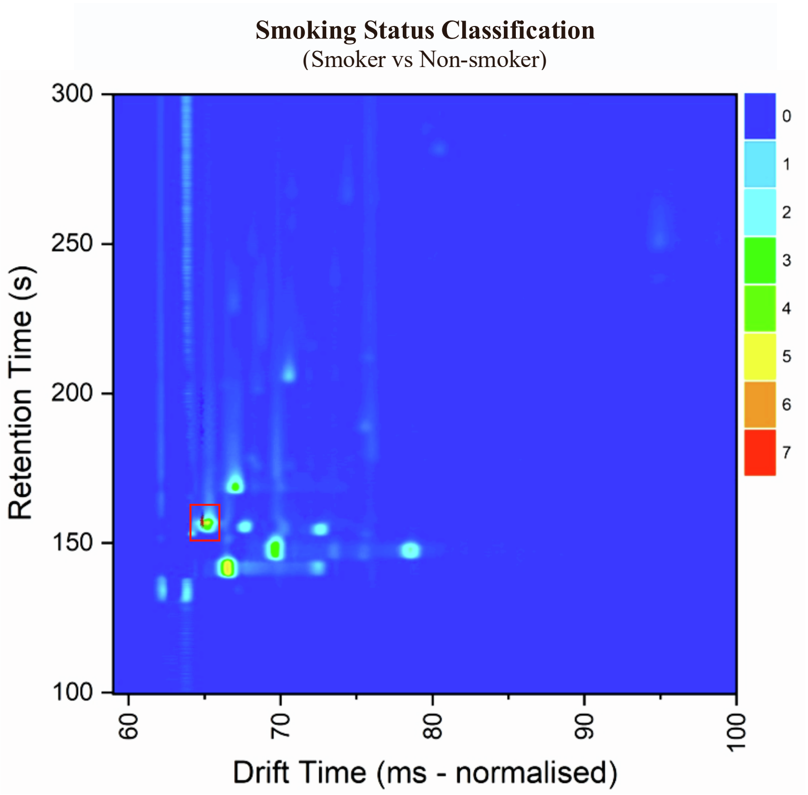

Supplementary Figure 4: An overlay of the discriminatory features established in the smoking status analyses onto designated topographic plots. The y-axis is the retention time obtained from the gas chromatography column and the x-axis is the drift time reported by the ion mobility spectrometer.
